# Television viewing and sleep are associated with overweight among urban and semi-urban South Indian children

**DOI:** 10.1186/1475-2891-6-25

**Published:** 2007-09-20

**Authors:** Rebecca Kuriyan, Swarnarekha Bhat, Tinku Thomas, Mario Vaz, Anura V Kurpad

**Affiliations:** 1 Division of Nutrition, St John's Research Institute, St John's National Academy of Health Sciences, Bangalore 560034, India; 2 Department of Paediatrics, St. John's National Academy of Health Sciences, Bangalore 560034, India; 3 Division of Public Health, St John's Research Institute, St John's National Academy of Health Sciences, Bangalore 560034, India

## Abstract

**Background:**

Childhood obesity is an emerging problem in urban Indian children and increases in childhood overweight and obesity may be major contributors to the adult obesity epidemic. Thus, identifying potential risk factors for childhood obesity and formulating early interventions is crucial in the management of the obesity epidemic. The present study was aimed at evaluating dietary and physical activity patterns as determinants of overweight in a sample of children.

**Methods:**

Five hundred and ninety eight children aged 6–16 years, visiting St. John's Medical College Hospital, Bangalore City, India for minor complaints or routine checkups were recruited into the study. These children were studied for their physical activity patterns, sleep duration, sedentary habits and eating behaviours as potential determinants of overweight.

**Results:**

Decreased duration of sleep and increased television viewing were significantly associated with overweight. Among the eating behaviours, increased consumption of fried foods was significantly associated with overweight.

**Conclusion:**

Our data suggests that duration of sleep, television viewing and consumption of fried foods may be significant factors that contribute to overweight. Further longitudinal studies are needed to confirm these findings.

## Findings

Childhood obesity is emerging as a major health problem in India, especially in children from urban higher socio-economic areas; for example, about 30% of children were overweight in an affluent Delhi school [1]. The adverse health effects of obesity in children justify the need to look for potential risk factors and provide suitable interventions. Factors contributing to childhood obesity, such as parental obesity, eating behaviours, TV viewing and lack of physical activity have been studied in Western settings [2,3]. Recent studies suggest that sleep duration is an important behavioural antecedent of obesity [4,5]. Indian studies are presently lacking with regard to the determinants of overweight/obesity among children. The aim of the present study was to evaluate dietary and physical activity patterns as determinants of overweight in a sample of children from different socioeconomic backgrounds, visiting a hospital for routine health checks or for minor complaints.

This was a cross-sectional, questionnaire based study on 598 otherwise healthy children (male and female) aged 6 – 16 years, recruited from children visiting St. John's Medical College Hospital, Bangalore, for vaccinations or minor complaints. None of the children had a history of recent weight loss. The study was approved by the institutional ethical review committee and parental informed consent was obtained.

Information was collected by a trained nutritionist from parents and, in older children, from themselves. Information included the date of birth, address, parent's occupation/income, habitual frequency and duration of different activities in school, games, travel, tuition, sedentary habits at home, household work, hobbies, exercise and sleep. The level of occupation, education and income of the parents were combined to arrive at an indicator for the socio-economic status of the child. The habitual frequency (per week) of consumption of chocolates, ice-creams, bakery foods, soft drinks, fried foods and non-vegetarian food, as well as the frequency of eating out, was obtained. Anthropometric measurements included body weight (nearest 0.1 kg) and height (0.1 cm), from which body mass index (BMI) (kg/m^2^) was calculated. In addition, waist and hip circumferences were measured according to a standardized protocol [6].

The children were classified as overweight or normal weight, based on their BMI [7]. Their habitual activities were grouped into 3 broad domains, consisting of sedentary activities (all sedentary activities at school and home), rigorous activities (physical training at school, games and exercise at and after school) and sleep. The duration of TV viewing was considered separately as an independent variable. Separate logistic regression analyses were performed to identify the significant associations of overweight with each of the habitual activity categories or eating behavior, while adjusting for age, gender, home location (urban or peri-urban) and socioeconomic status. Finally, associations between different significant predictors of overweight/obesity were also explored using cross tabulation and logistic regression with all three significant predictors (duration of sleep, duration of TV viewing and frequency of eating fried food) in the same model which examined the independent effect of all the three predictors and the interaction effect between them.

Urban children constituted 73% of the whole group, while the remaining children were from semi- urban areas (small towns). Fifty four percent (n = 324) of the children were male. The range of body weight was from 14 to 68 kg and the body mass index (BMI) ranged from 9.8 to 29.8 kg/m^2^. Their ages ranged from 6 to 16 years, and the children came from lower to middle socio economic status households. Based on their BMI  [7], 6.4% (n = 38) of the children were overweight.

The duration of sleep and TV viewing were significantly associated with overweight. Children who slept less than 8.5 hours/day had significantly higher odds (6.7, p = 0.013) of being overweight when compared to children who slept more than 9.5 hours/day, after adjustments for age, gender, location of stay and socioeconomic status. (Table 1). The adjusted odds of being overweight for children who viewed television for greater than or equal to 1.5 hours/day was 19.6 (p = 0.001), when compared to children who viewed television for less than or equal to 45 minutes/day (Table 1). Among eating behaviours, the consumption of fried food items, more than 6 times/week, was associated with significantly higher odds of being overweight (3.1, p = 0.014) when compared to fried food consumption less than 2.5 times/week. None of the other eating behaviours were found to be significantly associated with being overweight.

**Table 1:** Relationship between habitual activities/eating behaviours and overweight (OW)

Activity/Eating behaviour	Tertiles	n (%)	Median (IQR)	OR^1^	95% CI	p	OR^4^	95% CI
Sleep (hours/day)	≤ 8.5	203(33.9)	8 (7.5–8.5)	6.7	1.5, 30.2	0.013	6.8	1.4, 32.1
	8.51–9.5	257 (43)	9 (9–9.5)	3.6	0.8, 16.3	0.09	3.7	0.8, 17.4
	> 9.5	138 (23.1)	10 (10–10.5)	1	-	-	1	-
Sedentary activities^2 ^(hours/day)	≤ 7.4	200 (33.4)	6.7 (6.2–7.1)	1	-	-		
	7.41–8.9	199(33.3)	8.1(7.8–8.5)	0.6	0.3, 1.5	0.27	-	-
	> 8.91	199 (33.3)	10.2(9.5–11.2)	0.9	0.4,2.2	0.91		
Rigorous activities^3 ^(hours/day)	≤ 1.61	195 (32.6)	1 (0.6–1.4)	1.7	0.8, 3.8	0.20		
	1.62–2.61	203 (33.9)	2.0 (1.8–2.3)	0.8	0.3, 2.0	0.65	-	-
	> 2.61	200 (33.4)	3.4 (3–4.1)	1	-	-		
TV viewing (hours/day)	≤ 0.75	217 (36.3)	0.7 (0.5–0.75)	1	-	-	1	-
	0.76–1.5	266 (44.5)	1 (1–1.5)	3.1	0.8, 11.3	0.09	3.1	0.8, 11.4
	> 1.5	115 (19.2)	2 (2–3)	19.6	5.5, 69.4	< 0.001	19.8	5.4, 71.9
Fried foods (frequency/week)	≤ 2.5	199 (33.3)	1 (0.3–2.0)	1	-	-	1	-
	2.5–6	199 (33.3)	3.7 (3.0–4.7)	0.5	0.2, 1.6	0.27	0.4	0.1, 1.1
	> 6	200 (33.4)	10.8 (8.0–16.5)	3.1	1.3, 7.6	0.014	1.6	0.7, 3.9

IQR – interquartile range, OR- odds ratio, 95% CI – 95% Confidence Interval, p = level of significance.

^1 ^ORs were adjusted for age, gender, living location and socioeconomic status.

^2 ^-includes all sedentary activities at school and home

^3 ^-includes physical training at school, games at and after school and exercise

^4 ^-OR were adjusted for age, gender, living location and socioeconomic status and the other significant predictors of overweight in the data

Short sleep duration has been shown to be a risk factor for obesity in children [4,5], through the modulation of hormones such as leptin and ghrelin, the reduced levels of which can increase hunger and appetite and influence weight gain [8,9]. In this study, the duration of sleep was based on self reportage and this might represent an overestimate in absolute terms as sleep was reported as 'going to bed' and 'rising' rather than as actual sleep. However, it is unlikely that errors in self reportage would have contributed to a systematic error in between those who reported sleeping less or more. Children with television sets in their rooms, spent less time in bed on weekdays and reported higher overall levels of being tired [10-12]. It is likely that behaviours could cluster within individuals, and that increased television viewing could be associated with lower sleep duration. It was observed that the distribution of TV viewing in its tertiles was 34%, 45% and 21% in children who slept less than 8.5 hrs (lowest tertile of sleep). Hence, there was no significant clustering observed in the present study. It has also been recommended that the children's total media time (TV, video and video games) to be limited to no more than 1 to 2 hours of quality programming per day [13], however, it seems that even within this recommendation, there may be potential for a graded response towards weight gain with increasing duration of television viewing. None of the children in the present study played video games regularly.

With regard to diet, there is evidence of a demographic, epidemiological and nutrition transition in India particularly in the urban areas characterised by a shift in dietary patterns toward a higher fat and sugar content linked to an ongoing epidemic of chronic disease and obesity [14]. The results of the present study demonstrate that the increased consumption of fried and high fat foods were significantly associated with overweight. Similar results were seen in a previous study on children aged 9–14 years, where the higher consumption of fried foods from outside home was associated with greater total energy intakes, poorer diet quality and excessive weight gain [15]. In the present study again, there was no clustering effect observed between the high fat or junk food consumption and potentially unhealthy behaviours such as television viewing. The percentage distribution of TV viewing in its tertiles was 33%, 45% and 23% in the highest tertile of fried food consumption.

The findings of the present study suggest that behavioural modifications to increase sleep time, limit television viewing, as well as the limiting of consumption of fried foods may be useful for health promotion programs to prevent weight gain in childhood. Reducing television viewing to a minimum (1 to 1.5 hours/day) is desirable, as is encouraging children to sleep for at least 9 hours a day. Another simple message might be to reduce the frequency of eating fried and high fat foods to a minimum (2–3 times per week). Further longitudinal and dose response studies are however needed to confirm the potential link between these variables.

## Authors' contributions

RK was involved in the conception, design, acquisition of data, analysis and interpretation of data and writing of the manuscript.

SB was involved in conception, design and acquisition of data.

TT performed the statistical analysis.

MV was involved in drafting the manuscript and revising it critically for content.

AVK was involved in the conception, interpretation of data, drafting the manuscript and revising it for important intellectual content.
